# Regulatory role of PI3K/Akt/WNK1 signal pathway in mouse model of bone cancer pain

**DOI:** 10.1038/s41598-023-40182-w

**Published:** 2023-08-31

**Authors:** Xiao Fu, Yanhong Zhang, Rui Zhang

**Affiliations:** 1 Department of Anesthesiology, Taizhou Central Hospital (Taizhou University Hospital), Taizhou, 318000 China; 2 Inner Mongolia Medical University, Hohhot, 010110 China; 3 Department of Anesthesiology, Peking University Cancer Hospital Inner Mongolia Hospital/Cancer Hospital Affiliated to Inner Mongolia Medical University, Hohhot, 010020 China

**Keywords:** Bone cancer, Tumour-suppressor proteins

## Abstract

In the advanced stage of cancer, the pain caused by bone metastasis is unbearable, but the mechanism of bone cancer pain (BCP) is very complicated and remains unclear. In this study, we used 4T1 mouse breast cancer cells to establish a bone cancer pain model to study the mechanism of BCP. Then the paw withdrawal mechanical threshold (PWMT) and the hematoxylin-eosin staining were used to reflect the erosion of cancer cells on tibia tissue. We also determined the role of proinflammatory factors (TNF-α, IL-17, etc.) in BCP by the enzyme-linked immunosorbent assay in mouse serum. When GSK690693, a new Akt inhibitor, was given and the absence of intermediate signal dominated by Akt is found, pain may be relieved by blocking the transmission of pain signal and raising the PWMT. In addition, we also found that GSK690693 inhibited the phosphorylation of Akt protein, resulting in a significant decrease in with-nolysinekinases 1 (WNK1) expression in the spinal cord tissue. In the BCP model, we confirmed that GSK690693 has a relieving effect on BCP, which may play an analgesic effect through PI3K-WNK1 signal pathway. At the same time, there is a close relationship between inflammatory factors and PI3K-WNK1 signal pathway. The PI3K/Akt pathway in the dorsal horn of the mouse spinal cord activates the downstream WNK1 protein, which promotes the release of inflammatory cytokines, which leads to the formation of BCP in mice. Inhibition of Akt can reduce the levels of IL-17 and TNF-α, cut off the downstream WNK1 protein signal receiving pathway, increase the PWMT and relieve BCP in mice. To clarify the analgesic target of BCP, to provide reference and theoretical support for the clinical effective treatment of BCP and the development of new high-efficiency analgesics.

## Introduction

In recent years, with the rapid development of medical treatment technology, the survival rate of cancer patients has increased significantly, from 30.9% 10 years ago to 40.5%^1^. The extension of life span has increased the reproduction and infiltration time of cancer cells. At present, the proportion of cancer-induced bone metastasis is as high as 70–80%^2^. The primary manifestation of bone metastasis is pain or pathological fracture, which inevitably increases the incidence of bone cancer pain (BCP) and seriously affects the quality of life of patients^3^. At present, the clinical treatment measures (drug therapy, radiotherapy, chemotherapy, nerve block, surgical treatment, etc.) are still lack of specificity, and the management and treatment of BCP has not achieved satisfactory results.

BCP is a clinical disease in which cancer cells invade bone tissue and destroy bone structure to cause severe pain when the primary tumor develops to the middle and late stage. With the help of the matrix metalloproteinases (MMPs) produced by tumor cells, cancer cells activate fibroblasts and secretes vascular endothelial growth factor and chemokines, which are separated from primary tumors and enter the adjacent vascular lumen^4^. After entering the systemic circulation, tropomyosin receptor kinase B (TrkB) is overexpressed on the tumor cell membrane, which activates phosphatidylinositol 3-kinase (PI3K)-protein kinase B (Akt) signal pathway^5^, and up-regulates some cell surface proteins (CD47, etc.) to inhibit macrophage phagocytosis^6^. Finally, with the help of a variety of chemokines in the blood (CXCL10, CXCL12, CX3CL1 and CCL5, etc.)^7–10^ to adhere to the bone marrow endothelium, implant bone, the tumor cells settled in the bone will continue to grow and divide and destroy the bone structure. At the same time, there will be a large number of pro-inflammatory cytokines released (IL-1β, IL-6, TNF-α, etc.), they can sensitize or activate bone nerve sensory nerve endings, transmit nociceptive stimuli to the spinal cord, and finally receive perception in the brain, and the body shows severe pain^11^.

PI3K/Akt signal pathway plays an important role in the process of bone cancer pain caused by bone metastasis. PI3K phosphorylates the inositol cyclic 3ʹ-OH group in inositol phospholipids, and through the activation of receptor protein tyrosine kinase (RPTK), catalyzes the allosteric activation of the subunits to form the second messenger phosphatidylinositol-3, 4, 5-triphosphate (PIP3)^12^, which binds to the intracellular serine/threonine protein kinase Akt and regulate the phosphorylation of DNA-dependent protein kinase at Akt serine 473 and phosphatidylinositol-dependent kinase-1 at threonine 308^13–15^. The activated Akt is dissociated from the cell membrane and transferred to the cytoplasm or nucleus. And then continue to target the regulation of downstream signal molecules, participate in a variety of cell proliferation, migration, metabolism, protein synthesis and so on^16^. The increased expression of phosphorylated Akt leads to the sustained high expression of receptor activator of nuclear factor-κB receptor activator (RANK) in the precursor of osteoclasts, which plays a role in osteoclasts and osteoblasts. Inhibition of Akt can block the up-regulation of RANK expression and osteoclast formation^17^.

Tumor necrosis factor-alpha (TNF-α) is an osteoclast cytokine that can cause bone erosion. TNF-α directly promotes the expression of RANKL in osteocytes and stimulates the differentiation and maturation of osteoclasts^18^. It can also act on bone matrix and expose it to osteoclasts^19^. In addition, TNF-α enhanced the expression of sclerosin in osteocytes, which also promoted the formation of osteoclasts. In recent years, it has been found that interleulin-17 (IL-17) secreted by glial cells of dorsal root ganglion binds to neuronal IL-17 receptors and mediates hyperalgesia^20^. In addition, IL-17 is closely related to PI3K/Akt signal pathway, which induces increased expression of PI3K/Akt protein, promotes cancer cell migration and progression^21^, may protect tumor by promoting immune system-mediated tumor rejection^22^, and participates in the maintenance of bone cancer pain by activating P13K/Akt signal pathway^23^. Both TNF-α and IL-17 play an important role in the formation of bone cancer pain, but the mechanism of BCP has not been fully elucidated because of its complex mechanism.

The role of With-nolysine kinases 1 (WNK1) in tumorigenesis is partly achieved by stimulating tumor cell proliferation. WNK1 plays a key role in pathological nervous system signal transduction, phosphorylating inward Na^+^-K^+^-Cl^–^co-transporter protein 1 and extroverted Kumbai-co-transporter protein 2 through intermediate oxidative stress, changing ion concentration response to γ-aminobutyric acid to activate other silent pathways^24^. Kahle et al. established a specific WNK1-deficient HSN2 exon knockout mice and found that these mice were less sensitive to mechanical stimulation^25^. Some studies have found that the activation of Akt triggers WNK1-mediated lung cancer progression^26^. These studies suggest that WNK1 is involved in cancer progression and neuropathic pain regulation. Therefore, in this study, a new competitive Akt inhibitor of ATP, GSK690393, was used to block the Akt pathway to study the mechanism of PI3K/Akt/WNK1 in bone cancer pain. To identify the target of analgesia, to provide reference and theoretical support for the clinical effective treatment of bone cancer pain and the development of new and efficient analgesics.

## Results

### Effect of GSK690693 on paw withdrawal mechanical threshold in mice

**Figure 1:**
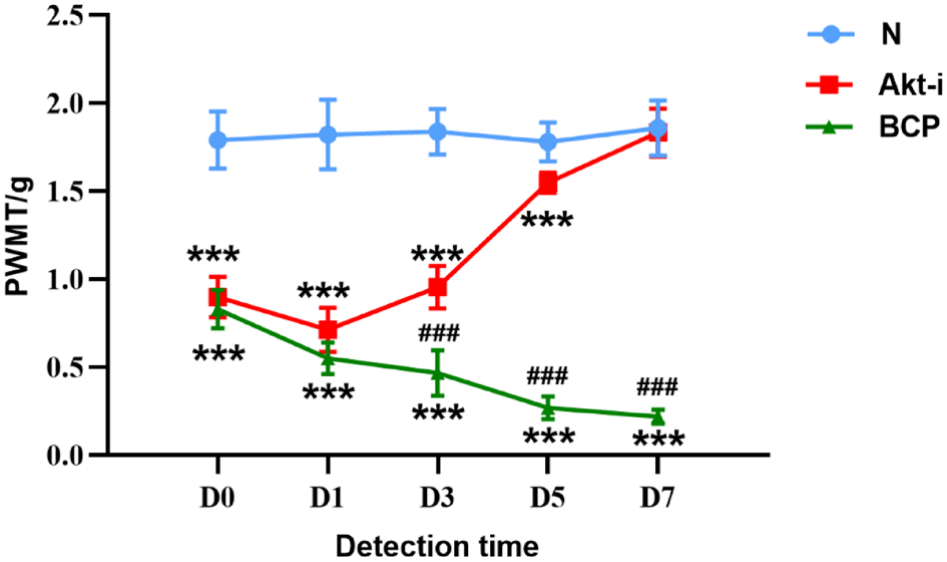
Comparison of PWMT among N group, BCP group and Akt-i group. n = 6. **P < 0.001 indicates that there is a statistically significant difference compared with N group, and ^***##***^*P* < 0.001 indicates that there is a statistically significant difference between BCP group and Akt-i group.

Fourteen days after inoculation of tumor cells, Akt-i group was treated with 20 mg/kg intraperitoneal injection of Akt inhibitor (GSK690693) for 5 times, once a day (2 days off for 2 days), and other groups were given the equal volume of solvent. At 30 min before the beginning of the experiment and on the 1st, 3rd, 5th and 7th day after the start of the experiment, PWMT was measured by Von Frey fiber pain meter 30 min after each administration or solvent, and the above time was recorded as D0, D1, D3, D5, D7, respectively. The change of PWMT in mice is shown in the Fig. 1.

We found that there was no significant difference in PWMT between the BCP group and the Akt-i group at D0 and D1 time points. PWMT in the BCP group and the Akt-i group was lower than that in the N group (*P* < 0.001). Cancer cells destroyed bone tissue and pain stimulation induced hypersensitivity in mice. As the cancer cells proliferated over time, destroying bone tissue and eroding the surrounding muscle tissue, the threshold of mechanical foot contraction in mice showed a downward trend (Fig. 1). From the third day, it was found that there was a difference between PWMT in the Akt-i group and the BCP group (*P* < 0.001). From the Fig. 1, it was obvious that the PWMT of mice in the Akt-i group showed an upward trend, and there was no significant difference in the last PWMT between the Akt-i group and the N group. The above results show that GSK690693 can increase PWMT and relieve BCP in mice.

### Hematoxylin-eosin staining

**Figure 2:**
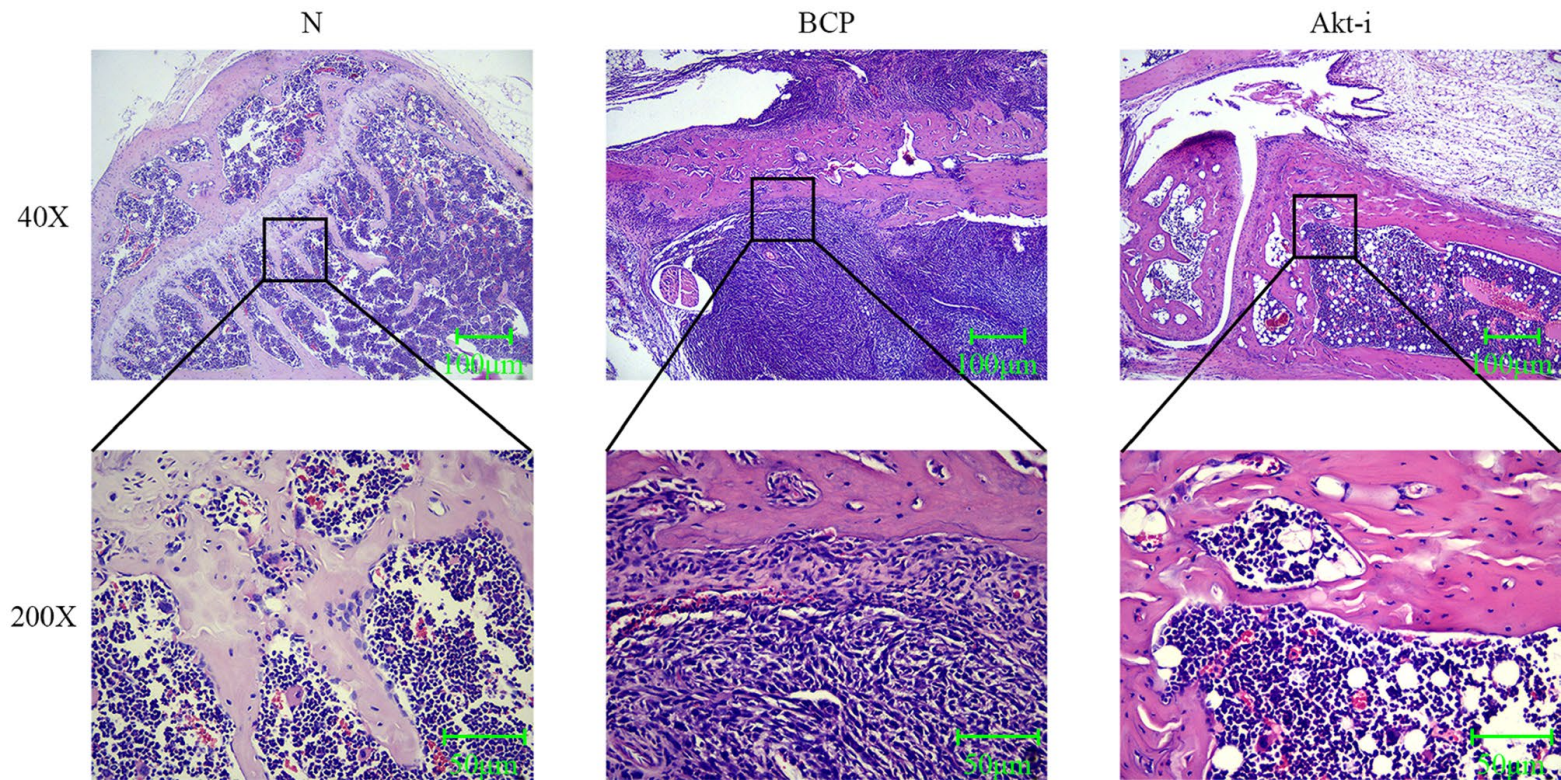
Hematoxylin-eosin staining of tibia in mice. N: Normal group, BCP: Bone cancer pain group, Akt-i: GSK690693 group. N: Bone tissue and bone marrow cavity intact. BCP: The cancer cells engulfed the bone trabeculae and destroyed the bone cortex. Akt-i: The growth of cancer cells stagnated and the destruction of bone marrow cavity was not obvious.

After GSK690693 injection, the tibia tissue of BCP mice was taken for pathological examination, and the pathological film could clearly see that the tibia tissue of BCP mice was completely eroded by cancer cells, engulfed the bone trabeculae, destroyed the bone cortex, and the cancer cells expanded continuously and infiltrated the surrounding muscle tissue. The pathological film of mice in the group N could clearly see bone trabeculae and normal bone marrow cells, showing that the bone marrow cavity of tibia was intact, no bone and trabecular destruction, and no tumor cell growth. In contrast, in the BCP group, the bone marrow cavity of tibia was destroyed obviously, and there was osteolytic destruction of bone and trabecula. At the same time, the bone marrow cavity of tibia was filled with a large number of tumor cells, and cancer cells could be seen infiltrating and growing outside the cavity through the destroyed bone marrow cavity. After injection of GSK690693, the bone marrow cancer cells obviously stagnated, the periosteal destruction was not obvious, the bone trabecular results were complete, and there was no tumor cell growth (Fig. 2).

### Expression of TNF-α, IL-17 and WNK1 in serum of mice

**Figure 3:**
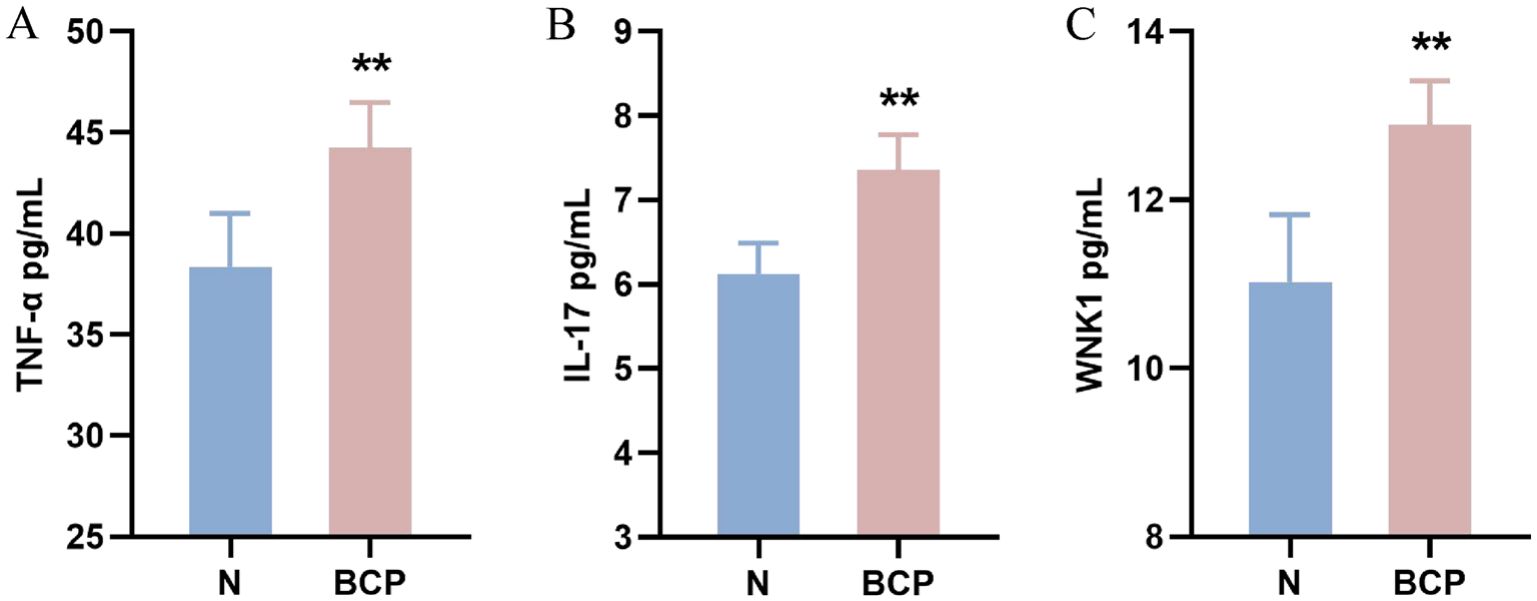
Expression of TNF-α, IL-17 and WNK1 in serum of mice after 14 days. n = 5; **P < 0.01, indicating that there is a difference in the expression level between BCP group and N group.

**Figure 4:**
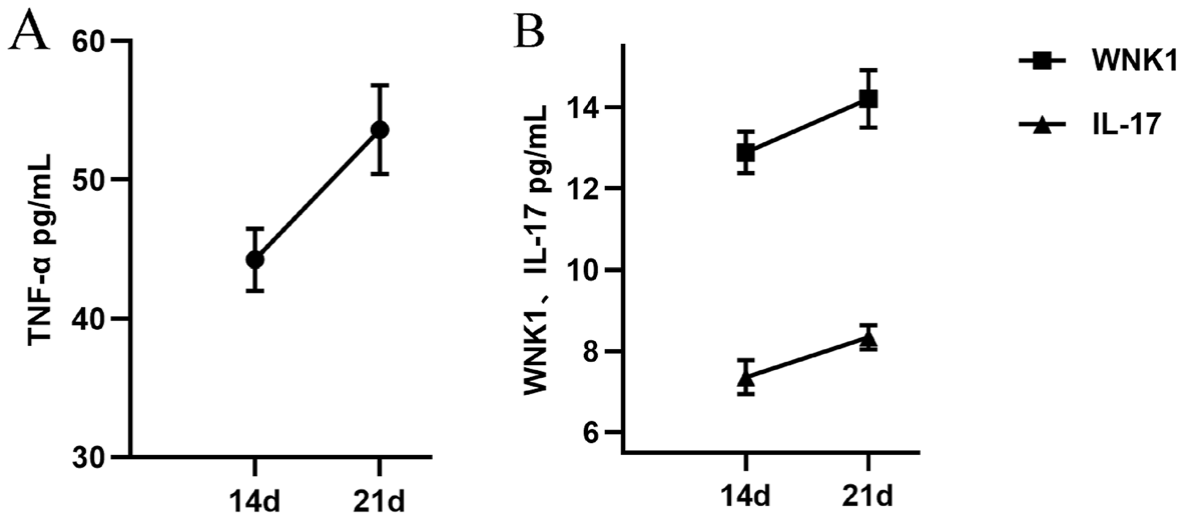
Change trend of TNF-α, IL-17 and WNK1 in serum of mice with time. n = 5; (**A**): The expression of TNF-α up-regulated with the passage of time. (**B**): WNK1 and IL-17 expressed the upward trend of horizontal range with the passage of time.

**Figure 5:**
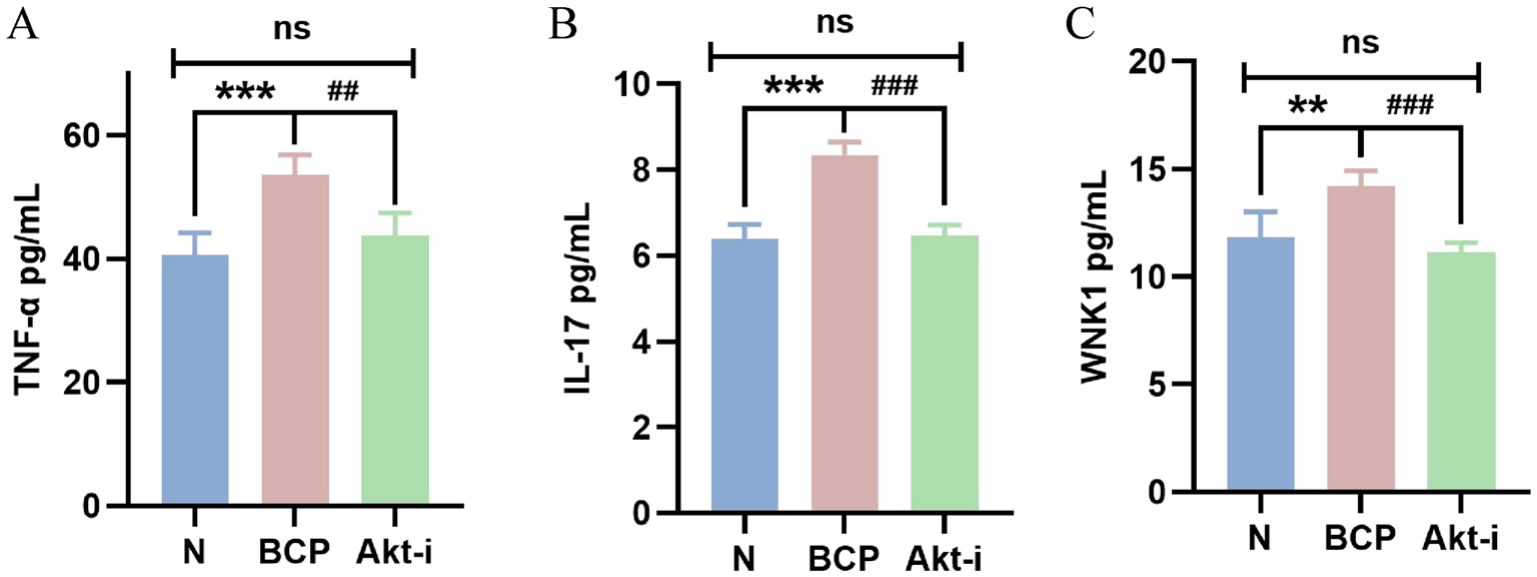
Expression of TNF-α, IL-17 and WNK1 in serum of mice after intraperitoneal injection of GSK690693. n = 5; **P < 0.01, ***P < 0.001, indicating that there is a difference in the expression level between BCP group and N group; ^***##***^*P* < 0.01, ^***###***^*P* < 0.001, indicating that there is a difference in expression level between Akt-i group and BCP group; ** and ^**##**^ are considered to be highly statistically significant, and *** and ^**###**^ are considered to be statistically significant.

The expression of TNF-α, IL-17 and WNK1 in serum of mice was detected by ELISA method. Fourteen days after inoculating 4T1 breast cancer tumor cells, blood was taken from the eyeballs of mice in the group N and the group BCP. After centrifugation, the supernatant was taken for ELISA detection. It was found that the expression of TNF-α in serum of mice with bone cancer pain was significantly higher than that of normal mice (*P* < 0.01) (Fig. 3A). And showed an upward trend with the passage of time (Fig. 4A), participating in the malignant process of bone cancer pain and promoting cell necrosis or apoptosis. When GSK690693 was given, we found that the expression of TNF- α decreased slightly (Fig. 5A).

We found that the expression of IL-17 in the serum of mice with bone cancer pain was up-regulated (Fig. 3B). Cancer cells stimulated the body to release a large number of inflammatory factors to participate in the formation of cancer pain, and showed an upward trend with time (Fig. 4). After administration of GSK690693, the expression of IL-17 decreased significantly (*P* < 0.001) (Fig. 5B), which may block the pain signal transmission and inhibit the inflammatory response.

In the serum of mice with bone cancer pain, the expression of WNK1 was significantly higher than that of normal mice (*P* < 0.01), and showed an upward trend with the passage of time (Fig. 4). After the last intraperitoneal injection of GSK690693, the orbital blood was taken within 2–4 h, and the supernatant was taken after centrifugation for ELISA detection. It was found that the expression level of WNK1 in serum of Akt-i group was significantly lower than that of BCP group (*P* < 0.001), but not significantly different from that of N group (Fig. 5C). It was found that the change of WNK1 expression may be related to PI3K/Akt signal pathway, down-regulating the expression of Akt, WNK1 was also inhibited, while the expression of inflammatory factors decreased.

### Expression of PI3K, Akt and WNK1 in mouse spinal cord

**Figure 6:**
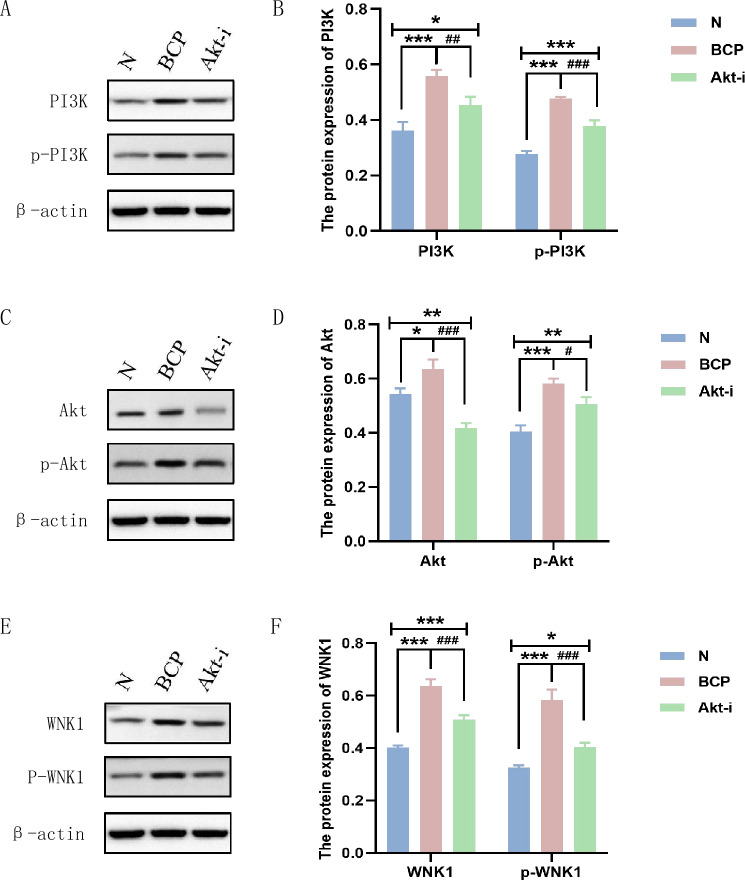
(**A**)、(**C**)、(**E**) Western blotting to detect the expression of PI3K, p-PI3K, Akt, p-Akt, WNK1 and p-WNK1 protein. (**B**)、(**D**)、(**F**) The optical density of protein bands detected by Western blotting was statistically quantified by IPP 6.0 software; (**B**) Protein expression of PI3K and p-PI3K; (**D**) Protein expression of Akt and p-Akt; (**F**) Protein expression of WNK1 and p-WNK1. N: Normal group, BCP: Bone cancer pain group, Akt-i: Akt inhibitor group. n = 3; *P < 0.05, **P < 0.01, ***P < 0.001, which means that compared with group N, the difference is statistically significant; ^***#***^*P* < 0.05, ^***##***^*P* < 0.01, ^***###***^*P* < 0.001, which means that compared with the BCP group, the difference was statistically significant; * and ^**#**^ are considered to be statistically significant; ** and ^**###**^ are considered to be highly statistically significant; *** and ^**###**^ are considered to be of great statistical significance.

Western blotting (WB) was used to detect the expression of PI3K, p-PI3K, Akt, p-Akt, WNK1 and p-WNK1 in L_4-6_ spinal cord. We found that the expression of p-PI3K and p-Akt in the spinal cord of mice with bone cancer pain was significantly increased (*P* < 0.001). And WNK1 was highly expressed in L_4-6_ segment of mouse spinal cord, and the expression of p-WNK1 in the BCP group was higher than that in the N group. When GSK690693 was given, the expression of p-Akt was significantly down-regulated. At the same time, it was found that the expression of WNK1 and p-WNK1 was also inhibited (Fig. 6), indicating that WNK1 is likely to participate in the activity of the body as a downstream signal molecule of PI3K/Akt. After administration of GSK690693, PWMT increased and pain relieved in mice (Fig. 1). These results suggest that PI3K/Akt-mediated WNK1 phosphorylation is involved in the occurrence and development of bone cancer pain.

## Discussion

In this experiment, 4T1 mouse breast cancer cells were successfully subcultured and a good activity and sufficient number of model cancer cells were obtained. The cancer cells were implanted into the tibial bone marrow of mice by operation, and the pain model of bone cancer in mice was established successfully. In the late stage of malignant tumor, bone metastasis is easy to occur, which destroys the normal structure and stable microenvironment of bone, and causes serious pain. In order to solve this clinical problem, some experts pointed out in 1939 that “mouse tumors are similar to human tumors in histogenesis, clinical process and histomorphology”^27^. Therefore, Schwei et al. injected NCTC2472 osteosarcoma cells into the femur to destroy periosteum, established a metastatic bone tumor model and studied the chemical changes of spinal cord and related dorsal root ganglia, which promoted the study of the mechanism of bone cancer pain. However, the success rate of this method is low (39%), and the modeling period is very long (21 days)^28^, which leads to a large error of the test results. In order to make up for the above problems, some researchers injected cancer cells into the calcaneus to establish the model of bone cancer pain, because this method is difficult to operate, it is difficult to choose a suitable puncture point, so it has not been widely used. With the increase of model research, it was found that the pain of tibial bone cancer pain animal model was similar to that of metastatic bone cancer pain in clinic^29^. Based on the above reasons, this experiment chose the tibial plateau as the testing point, where there are fewer nerves and vessels than the femur, and it is more convenient and quick to drill holes, which is not easy to damage animals and improve the postoperative prognosis of mice. And it can greatly improve the success rate of modeling and the life cycle of mice, which is more conducive to the smooth progress of experimental research.

There are many kinds of cancer cells in the model of bone cancer pain. Mouse breast cancer cell line 4T1 is a 6-thioguanine resistant cell line screened from 410.4 tumor lines without mutagen treatment^30^. In recent years, it has been used to establish the model of bone cancer pain. It has been found that 4T1 cells can spontaneously produce highly metastatic tumors in BALB-c mice and transfer to many organs (lung, liver and brain, etc.). At the same time, 4T1 cells will form initial lesions at the injection site. What is most noteworthy is that the growth and metastasis characteristics of 4T1 cells in mice are very similar to the occurrence and development of breast cancer in human body^31^. It was found that when the concentration of 4T1 cells was 1 × 10^7^ mL^−1^, the tumor formation rate was high and the growth was stable^32^. In this study, breast cancer cells of 4T1 mice were injected into tibial bone marrow to observe the tumor formation process of mice. The movements of lifting and licking feet increased gradually after 7 days, which was very similar to spontaneous pain in clinic. This model can be better used in the study of bone cancer pain.

The main findings of this study are: (1) Increased expression of p-PI3K/p-Akt/p-WNK1 signal pathway in spinal dorsal root ganglion of bone cancer mice; (2) Up-regulated expression of IL-17, TNF-α and WNK1 in mice with bone cancer pain; (3) Inhibition of Akt protein, decreased expression of WNK1 in blood and WNK1 in spinal dorsal root ganglion of mice with bone cancer pain. (4) Blocking PI3K-WNK1 signal pathway can reduce the hypersensitivity of mice with bone cancer pain to mechanical stimulation; (5) Blocking PI3K-WNK1 pathway can decrease the expression of IL-17 and TNF-α; (6) Blocking single PIC receptors (IL-17R and TNFR, etc.) in the brain may weaken the hypersensitivity of mice with bone cancer pain to mechanical stimulation by inhibiting PI3K-WNK1 signal.

In most pain syndromes, there are a variety of pain signal mechanisms around the site. Pain signals are transmitted through the peripheral nerve to the spinal cord and then to the thalamus and cortex of the brain, where pain is sensed, allowing us to locate the pain and describe its intensity. Akt is a widely expressed serine / threonine protein kinase, which is a cellular homologous gene of viral oncogene v-Akt^33^. It forms a PI3K/Akt signal pathway with its upstream protein PI3K and plays a very important role in neuropathic pain, inflammatory mechanical pain or thermosensitive pain and cancer pain^34–36^. Protein phosphorylation is the main mechanism of intracellular signal transduction and regulation^37^. Previous studies have found that when PDGFR β tyrosine Y740 and Y751 are phosphorylated, they bind to lipid kinase PI3K, phosphorylate the D3 position of the phosphoinositide ring, rapidly induce Akt kinase activity, and promote Akt phosphorylation^38^. During peripheral nerve injury and inflammatory pain, the level of phosphorylated Akt (p-Akt) in mouse DRG and spinal cord increased significantly and up-regulated in a time-dependent manner. The p-Akt was separated from the membrane and translocated into the nucleus, activating downstream signal molecules to participate in various cellular responses^39^. PI3K/Akt is usually up-regulated in pathological environment, but Li et al^40^. found that in the process of postoperative chronic pain, the expression of inflammatory factors (IL-1β, TNF-α, etc.) increased, while the expression of PI3K/Akt decreased. Microglia promote the transformation of astrocytes to A1 phenotype by reducing the activation of PI3K/Akt, which aggravates postoperative chronic pain. In this study, the pain signal regulation pathway of bone cancer was studied. We observed that the expression of p-PI3K and p-Akt was up-regulated in the spinal cord of BCP mice, and the expression of p-PI3K and p-Akt increased significantly after pain stimulation. In addition, we found that administration of Akt inhibitor GSK690693 could reduce mechanical hyperalgesia caused by bone cancer. Previous studies have shown that PI3K and PKB/Akt activation are also involved in chronic pain^41^. The above results suggest that PI3K/Akt plays different roles in the process of pain formation by activating a variety of downstream signal pathways.

WNK1 is a new serine / threonine protein kinase family discovered by Xu et al^42^. in the screening and identification of new members of the MAP kinase (MAP2K) family. WNK1 was early thought to be a blood pressure regulator and can regulate angiogenesis and induce cell migration in a range of cancer types^43^. In recent years, more and more evidence shows that WNK1 is a key kinase involved in many types of cancer, promoting cell proliferation and inducing cancer through anti-apoptosis and promoting survival function. By analyzing mouse models and patient data, the researchers found that WNK1 is one of the few genes uniquely associated with invasive breast cancer^44^. The upregulation of WNK1 protein can lead to the activation of its downstream pathway and the increase of potential cancer promotion. The fusion gene WNK1-ROS1 has been described as a new driver of lung adenocarcinoma^45^. In lung cancer cells and mouse tumor models, inhibiting the expression of WNK1 can reduce the expression of N-cadherin and smooth muscle actin induced by secreted protein acidic and rich in cysteine (SPARC). After down-regulation of WNK1 expression, the migration ability of non-small cell lung cancer cell lines CL1-5 and H1299 was inhibited^46^. WNK1 inhibits the activation of NLRP3 by balancing intracellular Cl^-^ and K^+^ concentrations during activation^47^. Knockout of WNK1 in primary glioma cells results in decreased phosphorylation of Na^+^-K^+^-2Cl^-^ cotransporter1 (NKCC1) and reduced glioma cell migration^48^. It has been found that the overexpression of Akt leads to the increase of WNK1 protein phosphorylation in the bone marrow microenvironment of multiple myeloma cells^49^. And the expression of WNK1 in bone cancer increased with time (Fig. 3). Insulin-like growth factors 1 (IGF-1) stimulation up-regulated Akt phosphorylation in WNK1 knockdown cells and induced autophagy through Akt/WNK1 pathway^50^. It is worth noting that the PI3K/Akt signal pathway is related to WNK1 in the detection of spinal cord tissue protein level in this experiment.The p-PI3K, p-Akt and p-WNK1 are co-expressed in spinal cord tissue, and the expression of p-Akt and p-WNK1 is up-regulated after pain stimulation, but decreases after the inhibition of Akt inhibitor GSK690693. PI3K-WNK1 pathway is a signal pathway that can not be ignored in the process of tumorigenesis. With the increase of the number of GSK690693 administration, the PWMT of mice in the Akt-i group gradually approached the mean value of PWMT in normal mice. Down-regulation of WNK1 by small interference can reduce mechanical hyperalgesia and exercise-induced pain^51^. However, some studies have shown that WNK1 is an essential gene in mice, and knockout of mouse WNK1 gene will inhibit the development of functional vascular structure, resulting in embryonic death in more than ten days. The absence of WNK1 also prevents the migration and angiogenesis of primary cultured human endothelial cells^52^. Inhibition of Akt can inactivate the PI3K/Akt/WNK1 pathway, thus change the level of pro-inflammatory factors in the inflammatory microenvironment, and finally relieve chronic tibial cancer pain. Whether other mediators are involved in the regulation of this pathway is a matter of concern in the follow-up research. Therefore, it is very important to accurately find the activation of WNK1 upstream and downstream target signal molecules under bone cancer pain stimulation, which is the focus of the next experimental research.

Figure 1 shows that after 5 injections of GSK690693, PWMT was effectively restored and the expression of pro-inflammatory cytokines (PICs) was reduced, suggesting that PI3K-WNK1 pathway may regulate the inflammatory microenvironment and participate in chronic tibial cancer pain. In the process of non-small cell bone metastasis, the activity of tumor secretory factors (such as IL-1, IL-6 and TNF-α) leads to excessive formation of osteoclasts, induces mobilization of bone marrow-derived cells that promote angiogenesis and tumor homing, and finally promotes tumor growth^53^. Studies have shown that PICs mediators are present in midbrain periaqueductal gray (PAG), and the activation of PICs in PAG plays an important role in regulating pain response^54^. In the spinal nerve ligation model, the number of CD4^+^ T cells and T helper 17 cells in the spinal cord increased, accompanied by a significant up-regulation of IL-17^55^. IL-17 and IL-17R in the peripheral nervous system may also mediate glial-neuronal interaction^56^. In particular, IL-17 has been repeatedly demonstrated to indirectly lead to chronic pain by promoting immune cells to penetrate into damaged tissues, releasing nociceptive inflammatory factors or regulating synaptic transmission by directly acting on neurons^56–59^. The hypersensitivity of IL-17 knockout mice was alleviated, and the expression levels of proinflammatory cytokines such as TNF-α, IL-6 and interferon-γ were also decreased. It is well known that TNF-α is also closely related to chronic pain^60^. Fazzari et al^61^. found that the level of IL-17 was negatively correlated with the clinical pain score of cancer patients. In the serum of mice with bone cancer pain, IL-17 increased significantly and showed an upward trend with time. The high expression of IL-17 decreased the threshold of mechanical foot withdrawal in mice, and there was a negative correlation between them (Figs. 1 and 2B). After administration of GSK690693, the level of IL-17 gradually returned to normal (Fig. 4), which may indicate that the expression of IL-17 is regulated by PI3K-WNK1 signal pathway. In this process, PWMT is also close to normal mice, which further indicates that blocking a single PIC receptor in the brain (IL-17R and TNFR, etc.) may weaken the hypersensitivity of bone cancer pain mice to mechanical stimulation by inhibiting PI3K-WNK1 signal.

In this experiment, by observing the indexes of PWMT, ELISA and WB, it was found that GSK690693 could relieve the chronic cancer pain of tibia in mice. The expression of PICs in the serum of mice with bone cancer pain was time-dependent and closely related to the expression of PI3K, Akt and WNK1 protein. The PI3K/Akt pathway in the spinal dorsal horn of mice activates downstream WNK1, promotes the formation of bone cancer pain by up-regulating the level of inflammatory factors, inhibits Akt and blocks PI3K-WNK1 signal pathway, which can reduce the expression level of IL-17,TNF-α, increase the threshold of mechanical foot withdrawal and relieve bone cancer pain.

## Methods and methods

### Experimental animal

All experiments were conducted according to the guidelines of the International Association for pain Research^62^. BALB/c mice, 6–8 weeks old, weighing 18–22 g, female, clean grade, healthy, 36, were purchased from Beijing Huafukang Biotechnology Co., Ltd. Laboratory animal license number: SCXK (Beijing) 2019–0008. They were raised in separate cages in the laboratory of the experimental animal center. The temperature is 20–24 °C and the humidity is 40% murine 70%. Before the formal experiment begins, the mice freely ingest sufficient feed and drinking water in a quiet environment with natural circadian regularity, and feed adaptively for 5 days. The eyeballs of the mice were taken blood on the 14th day and 21st day respectively, and then the segments of L_4-6_ spinal cord were dissected and the tibia and muscle tissue were stripped off.

The animal experiment in this study was approved for implementation by the Kangtai Medical Animal Experiment Ethics Committee, and the study is reported in accordance with ARRIVE guidelines.

### Reagent

Isoflurane (G45880) was purchased from Beijing Yizejia Technology Co., Ltd. 4T1 cell (mouse breast cancer cell) CL-0007 was purchased from Wuhan Punosai Life Technology Co., Ltd. GSK690693 (HY-10249) is purchased from Merck Drugs & Biotechnology. TNF- α kit (CK-EN20852) and IL-17 kit (CK-EN20170) were purchased from Quanzhou Jiubang Biotechnology Co., Ltd. The WNK1 kit (YJ831821) was purchased from enzyme-linked organisms. Akt (AF6261), p-Akt (AF0832), PI3K (AF6241), p-PI3K (AF3242), WNK1 (AF6483) and p-WNK1 (AF3483) are purchased from Affinity bioscience.

### Cell culture

The purchased mouse 4T1 breast cancer cell suspension was thawed and added to the preheated RPMI-1640 complete medium containing 10% fetal bovine serum (FBS). The cells were cultured in 37 ℃ and 5% CO_2_ incubator, and the cells were adherent to the growth state. The number of cells was amplified by subculture, and when the cells grew to about 80%, 1–2 mL trypsin containing EDTA was added to the culture flask to digest the cells. when most of the cells began to become round and fall off, quickly tap the culture bottle or flip the culture bottle, and immediately add a complete medium containing more than 10% serum of 5 mL to terminate digestion. The cell concentration was counted and adjusted to 1 × 10^7^ cells/mL.

### BCP model

The mouse model of bone cancer pain was established according to Hansen et al^63^: the mice were placed on the operating table of ultra-clean animals. After ensuring that the pipeline of the anesthesia machine is complete and airtight, adjust the induction concentration of isoflurane to 3%, wait for complete anesthesia (gently shake the induction box without reaction), adjust the concentration of isoflurane to 1% for anesthesia maintenance, and start the model operation. The skin of the right limb was prepared, iodine was sterilized twice and deiodinated. Determine the plane of the tibia, cut the skin surface with a sterile scalpel and bluntly separate the subcutaneous tissue to avoid damage to blood vessels and nerves. Expose the joint, make a small opening from the tibial plateau joint with a 1 mL syringe needle, use a 50 μL microsyringe to enter the bone marrow along the hole, and then slowly inject 10 μL 1 × 10^7^ cells/mlmouse breast cancer cell suspension. The wound was sealed with bone wax and disinfected by iodophor. Suture the skin layer by layer, and disinfect the skin surface with iodophor again. After the operation, the mice were kept breathing in pure oxygen for about 5 min. The mice were placed in the incubator and waited for the mice to change from anaesthesia to wakefulness. The mice were observed in separate cages for 3 days. It was observed that the mice were reared in cages after natural exercise, no lameness and no abnormal activity. Antibiotics were given once a day three days after operation to prevent postoperative infection. The same volume of inactivated cells was injected into the right tibia of mice in the Sham group, and the rest of the operation was the same as that of the mice with bone cancer pain.

### Tumor monitoring and verification of bone cancer pain model

After the cultured mouse breast cancer cell line 4T1 (1 × 10^7^ cells/mL) was injected into the tibial joint (10 μL), the tumor growth of the right hindlimb was observed twice a day. On the 6th day after operation, subcutaneous tumor formation could be seen in the tibial plateau near the femur, and did not affect the health status of mice. The subcutaneous tumor was the most obvious on the 14th day after operation, and the maximum tumor size was between 1.8 and 2.0 cm. The PWMT of mice was measured with Von Frey fiber before 30 min and on the 1st, 3rd, 6th, 10th and 14th day after operation, which were recorded as D0, D1, D3, D6, D10, D14. On the 14th day after operation, the tibia was decalcified and the pathology was analyzed by HE.

### Detection of PWMT

In this experiment, the 50% PWMT of mice with bone cancer pain was detected by “Up-and-Down” method^64^. The whole process of the experiment was completed in the operation room of animal experiment, the room temperature was 20–25 °C, the air humidity was about 60%, and the operating time was 8:00–12:00. The PWMT of mice was measured 30 min before the beginning of the experiment and on the 1st, 3rd, 5th and 7th day after each administration or solvent, which was recorded as D0, D1, D3, D5, D7. Before each test, the mice were placed in a transparent plastic box with a wire mesh base (25 cm × 10 cm × 15 cm) and allowed to move freely for 20 min to adapt to the wire mesh platform. When the mice were quiet (the limbs did not move frequently), a series of Von Frey filaments (0.07,0.16,0.4,0.6,1.0,1.4,2.0,4.0 g) were taken to stimulate the central skin of the right foot of the mice. The filaments should be bent to “C” or “S” type and maintained for 6–8 s. The foot withdrawal reaction of the mice was observed and recorded according to the “Up-and-Down” method. A response (shrinking, walking, twitching or licking the foot) was recorded as a positive reaction “X” and no response as a negative reaction “O”. When the mice showed negative reaction, the center of the foot of the mice was stimulated with Von Frey filaments of large strength, and when the mice showed positive reaction, the stimulation of Von Frey filaments of small strength was used. The interval between the two stimuli was 3 min. When the experiment recorded the first time from positive reaction to negative reaction or negative reaction to positive reaction (“OX” or “XO” ride), four consecutive measurements were made to get a sequence combined with “O” or “X”. The sequence and the last test wire specification were input into the formula (1) to calculate the PWMT of the mouse hind paw.

If starting from 0.6 g, the animal has four consecutive positive reactions (that is, up to 0.07 g animals still have foot withdrawal, walking, twitching or licking reaction) or five consecutive negative reactions (that is, no foot withdrawal, walking, twitching or licking reaction in 4 g animals), 0.6 g or 4.0 g can be directly used as the 50% paw shrinking threshold in mice. If riding reaction occurs, but the minimum or maximum filaments are tested less than 4 times, continue to use the minimum or maximum filaments according to the “Up-and-Down” measurement record to rule out the possibility of false positive or false negative^65^.

The formula for calculating the response threshold of 50% mechanical foot withdrawal is as follows:

1 $$50\% \,{\text{PWMT}}\left( {\text{g}} \right) = 10^{{[{\text{Xf}} + {\text{k}}\delta ]}} /10000$$

Xf is the number of the last test wire, and δ is the average difference of the logarithm of each fiber, which is about 0.247 and κ is the coefficient value obtained by looking up the table of “X” and “O” sequences obtained according to the measurement.

### Indication at the end of the experiment

The mechanical threshold of paw withdrawal increased in the Akt-i group, severe claudication and decreased vital signs occurred in the bone cancer pain group, and the cervical dislocation ended the experiment. Euthanasia was carried out by cervical vertebra dislocation, and the death of mice was proved by touching mice without breath and pulse.

### Histopathological examination of tibia

The right hind limb of the mouse was cut off, the bone tissue and muscle tissue were stripped off, and the tibia of the mouse was fixed in 4% paraformaldehyde for 48 h. The fixed tissue was then transferred into EDTA decalcification solution for 15–30 days until the bone tissue softened. After washing the tissue with running water for 4 h, the tissue was dehydrated for 2 h with 80%, 90%, 95% and 100% ethanol in turn. Then transparent (placed in the same mixture of pure alcohol and xylene, xylene I and xylene II for 30 min, respectively), wax penetration (put in the mixture (xylene and paraffin) for 15 min, and then put into paraffin I for 6 min. Finally, overnight in paraffin II), embedding, slicing (4 μm thick), laying, pasting, baking and dewaxing (10 min each in xylene I and xylene II. Then put in anhydrous ethanol I and anhydrous ethanol II for 5 min each, and finally pass 95%, 90%, 80% and 70% alcohol for 5 min respectively). Then nuclear staining (slices were placed in hematoxylin dye solution for 30–60 s, then washed with pure water, differentiated with 1% hydrochloric acid alcohol for several seconds, 0.6% ammonia water returned to blue, rinsed with running water), cytoplasmic staining (sections stained in eosin dye solution, once stained immediately with distilled water, 75% alcohol differentiation when the staining color is heavy), dehydrated and sealed. Finally, the microscope linked to the computer is used to observe and analyze the pictures, and the appropriate multiple is selected to take pictures.

### Using ELISA to detect the expression level of TNF-α and IL-17

The standard samples with different concentration gradients were added to the standard hole (50 μL), and then the mouse serum sample hole was added to the sample hole (10 μL), and then 40 μL sample diluent was added to the sample hole. 100 μL horseradish peroxidase (HRP) labeled antibodies were added respectively. Samples and enzyme-labeled reagents were not added to the blank hole. After incubating the sealed lath in a 37 °C incubator for 60 min, gently pour out the liquid from the lath hole and pat dry on the absorbent paper. Then add flushing solution to each hole (diluted at 1:20), rest for 20 s, pour out, pat dry, and repeat washing plate 5 times. Add substrate developer A and developer B in each hole in order to avoid light and incubate for about 10 min after the standard sample appears obvious color gradient, and then add the terminating solution to terminate the reaction. Put the lath into the enzyme labeling machine, adjust the wavelength to 450 nm, record the absorbance (OD value), calculate the sample concentration, and detect the expression of TNF-α and IL-17 in mouse serum.

### Using ELISA to detect the expression level of WNK1

According to the instructions, the standard samples with different concentration gradients are prepared by using the original standard. The standard samples with different concentration gradients were added to the standard hole (50 μL), and then the mouse serum sample hole was added to the sample hole (10 μL), and then 40 μL sample diluent was added to the sample hole. 50 μL horseradish peroxidase (HRP) labeled antibodies were added respectively. Samples and enzyme-labeled reagents were not added to the blank hole. The sealed lath was incubated in a 37 °C incubator for 30 min. Gently pour out the liquid from the lath hole and pat dry on the absorbent paper. Then add flushing solution to each hole (diluted at 1:20), rest for 20 s, pour out, pat dry, and repeat washing plate 5 times. Add chromogenic agent An and chromogenic agent B in turn in each hole, avoid light and develop color for about 10 min, and then add terminating solution to terminate the reaction after the standard sample has obvious color gradient. Put the lath into the enzyme labeling machine, adjust the wavelength to 450 nm, record the absorbance (OD value), calculate the sample concentration, and detect the expression of WNK1 in mouse serum.

### Using WB to detect the expression level of PI3K/p-PI3K, Akt/p-Akt, WNK1/p-WNK1

The segmental tissue of L_4-6_ spinal cord of mice was put into 5 ml centrifuge tube, and 5 times volume of cell lysate (Western and IP cell lysate P0013) was added to each centrifuge tube. Then add a grinding steel ball and put it into a grinder (70 Hz for 60 s) and grind it 2–3 times. Protein was extracted by centrifugation 15 min (4 °C, 12,000 rpm). Then use the standard sample to determine the protein concentration and start electrophoresis, prepare 10% separate gel 8 mL and quickly pour it into the gel glass tank and wait for the gel to solidify. Pour out isobutanol, rinse the top surface of the gel with a buffer, dry it with absorbent paper, add 5% concentrated glue, insert the sample comb, and continue to gel at room temperature. Add the sample to the gel hole and connect the power supply, under the condition that the voltage is about 8 v/cm, through the concentrated gel and the separation gel, the 120 V high voltage constant voltage electrophoresis is used to make the dye to the appropriate position of the separation gel, and the end time of electrophoresis is determined by pre-dyeing protein marker. Prepare NC film transfer film, connect power supply, 100 V constant voltage transfer film for 90 min. The antibody was incubated, closed at first, and then incubated with the primary antibody reaction (1:300). After being fully washed, the membrane was incubated with horseradish peroxidase labeled anti-rabbit second antibody (1:300), and the immune reaction was observed. The excess liquid on the film was absorbed by filter paper and put into chemiluminescence imaging system (ChemiScope 6100). Finally, the band was quantified by Image Pro Plus 6.0 and normalized with *β*-actin as internal reference protein. PI3K/Akt/WNK1 and p-PI3K/p-Akt/p-WNK1 were detected by standard Western blotting. Membrane and first antibody Akt (AF6261), p-Akt (AF0832), PI3K (AF6241), p-PI3K (AF3242), WNK1 (AF6483) and p-WNK1 (AF3483) were purchased from GVS and Affinity bioscience.

### Statistical analyses

The data are presented as means ± SD. In order to compare these groups, one-way ANOVA was used to compare PWMT, ELISA and WB among the three groups, and Tukey method was used to test the pairwise comparison between groups. The test level α = 0.05, *P* < 0.05 indicates that the difference is statistically significant. The collection, arrangement, input and analysis of the data were analyzed by IBM SPSS 26.0 statistical software.

### Ethics approval

The animal experiment in this study was approved by the Kangtai Medical Animal Experimental Ethics Committee (Building 7, Peptide Valley Biopharmaceutical Industry Park, emerging Industrial demonstration Zone, Gu’an County, Langfang City, Hebei Province); Approval number: MDL2022-06-04-03.

## Data Availability

The data that support the findings of this study are available from the corresponding author upon reasonable request.
